# Analytics for Metabolic Engineering

**DOI:** 10.3389/fbioe.2015.00135

**Published:** 2015-09-07

**Authors:** Christopher J. Petzold, Leanne Jade G. Chan, Melissa Nhan, Paul D. Adams

**Affiliations:** ^1^Joint BioEnergy Institute, Physical Biosciences Division, Lawrence Berkeley National Laboratory, Berkeley, CA, USA; ^2^Department of Bioengineering, University of California Berkeley, Berkeley, CA, USA

**Keywords:** metabolic engineering, RNA-seq, proteomics, metabolomics, high-throughput screening, microfluidics

## Abstract

Realizing the promise of metabolic engineering has been slowed by challenges related to moving beyond proof-of-concept examples to robust and economically viable systems. Key to advancing metabolic engineering beyond trial-and-error research is access to parts with well-defined performance metrics that can be readily applied in vastly different contexts with predictable effects. As the field now stands, research depends greatly on analytical tools that assay target molecules, transcripts, proteins, and metabolites across different hosts and pathways. Screening technologies yield specific information for many thousands of strain variants, while deep omics analysis provides a systems-level view of the cell factory. Efforts focused on a combination of these analyses yield quantitative information of dynamic processes between parts and the host chassis that drive the next engineering steps. Overall, the data generated from these types of assays aid better decision-making at the design and strain construction stages to speed progress in metabolic engineering research.

## Introduction

**Figure 1:**
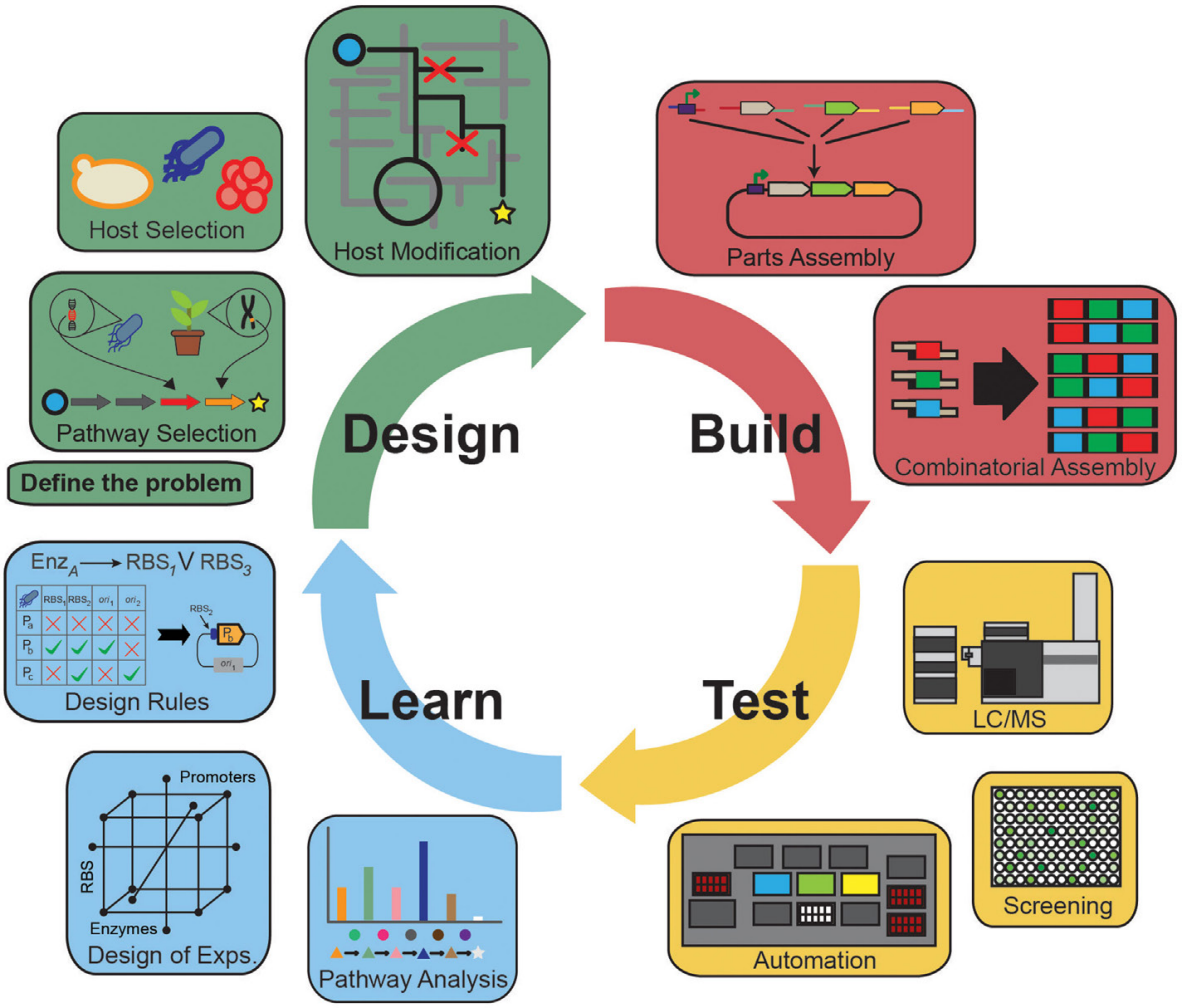
**The design–build–test–learn cycle of metabolic engineering highlighting important parts of each of the components**. The Design component identifies the problem, selects the desired pathway and host; the Build component selects, synthesizes, and assembles parts for incorporation into the host; the Test component validates the engineered strains for target molecule production, transcripts, proteins, and metabolites; the Learn component analyzes the Test data and informs subsequent iterations of the cycle.

Over the past decade, the field of metabolic engineering has realized several significant ­achievements centered on production of bulk (Atsumi et al., 2008; Saxena et al., 2009; Yim et al., 2011; Jarboe et al., 2012), high-value (Yang et al., 2013; Zhang and Stephanopoulos, 2013), and therapeutic compounds (Paddon et al., 2013). Stemming from these efforts have been transformational developments in the design–build–test–learn (DBTL) paradigm to accelerate pathway discovery, construction, characterization, and understanding. Innovative tools in each component of the cycle are propelling the field from the artisanal/single researcher model of science toward engineering principles of standardization, parameterization, and robust operation (Figure 1). Yet, attempts to generalize knowledge from earlier studies have not led to rapid progress toward these goals. As a result, successful integration of each component into a coherent whole that can rapidly inform subsequent engineering efforts is still a challenging endeavor. True paradigm-shifting advancement in metabolic engineering requires seamless workflows that are capability matched across all components to inform subsequent cycles.

Until recently, strain design was often treated as a one-off process, relying heavily on domain expertise with no standardization. Now, many software platforms that integrate and automate design parameters, enable strain construction, and incorporate knowledge from past experiments are readily available (Kelwick et al., 2014). A variety of novel computational tools are available to identify biosynthetic gene clusters (Hatzimanikatis et al., 2005; Campodonico et al., 2014; Medema et al., 2014; Weber et al., 2015), select pathways based on retrosynthesis of products (Hatzimanikatis et al., 2005; Campodonico et al., 2014; Carbonell et al., 2014a,b) or retro-fit enzymes to engineered pathways (Brunk et al., 2012). Likewise, genome-scale models (Feist and Palsson, 2008; Schellenberger et al., 2011; McCloskey et al., 2013) can identify beneficial host modifications *in silico* to prioritize changes based on factors related to target molecule production.

Once the pathway is chosen, there remain many questions related to which specific enzymes and how much of them are optimal, as well as which expression system should be used to achieve balanced protein levels. Consequently, a significant amount of research has focused on characterization of libraries of promoters (Alper et al., 2005; Anderson et al., 2010; Babiskin and Smolke, 2011; Davis et al., 2011; Mutalik et al., 2013) and terminators (Chen et al., 2013), as well as develop tools to predict ribosome-binding strength (Salis et al., 2009) or control protein location in the cell (Dueber et al., 2009). New technologies have advanced the Build component via low cost gene synthesis, rapid genome modifications [e.g., CAS9/CRISPR (Jinek et al., 2012)], and facile methods to rationally generate strain diversity [i.e., multiplex automated genome engineering (MAGE) (Wang et al., 2009) and trackable multiplex recombineering (TRMR) (Warner et al., 2010)]. As the scope of strain construction has grown, combinatorial approaches, like multivariate modular metabolic engineering (Ajikumar et al., 2010), have been applied to various parts to rapidly balance flux through the pathway (Latimer et al., 2014; Smanski et al., 2014). Yet, knowledge from previous work is infrequently predictive for new pathways, genes, and gene expression levels (Cardinale and Arkin, 2012; Kittleson et al., 2012). This problem is compounded when multiple genetic circuits or pathways are combined into a single organism often leading to unintended consequences. Thus, massive over construction of a pathway is required to find the best strain.

As a result, large-scale analysis of the engineered organisms is needed from the Test component. Yet, it lags far behind the recent Design- and Build-related advancements especially with respect to throughput, robustness, and generalizability. High-throughput assays, such as screens or selections that assay the target molecule, are ideally suited to strain optimization efforts, yet fail to provide sufficient information to efficiently identify pathway bottlenecks. Alternatively, detailed analyses of transcripts, proteins, and metabolites to query strain function can provide a rich dataset to identify bottlenecks that inform subsequent strain design, albeit for only a tiny fraction of strains that can be built. Detailed analyses of strain function are needed because engineering efforts often disrupt native cell processes that compete with the intended result. It is currently possible to analyze only a tiny fraction of the engineered strains for detailed omics analysis and quantification. The net result is a significant capability gap between the Design and Build components and that of the Test component of the DBTL cycle.

Unfortunately, learning is possibly the most weakly supported step in current DBTL cycle. Learn efforts to generalize knowledge from past experiments to inform design and build process decisions with the explicit goal of increasing the rate of successful outcomes are frequently limited. Many failure modes are possible and they are often difficult to identify and alleviate. Successful learning stems from observations from multiple iterations of the cycle, including analysis of failure modes at multiple functional levels (i.e., transcripts, proteins, metabolites). From this knowledge, improved design rules for assembling biological systems with predictable behavior can be created. With the Design and Build capabilities outpacing Test developments metabolic engineers face distinct difficulties making significant progress. This lack of actionable data prevents meaningful learning from past efforts. This review will detail the common analytical techniques to enable characterization of a broad range of components and their interactions.

## Target Molecule Detection

**Figure 2:**
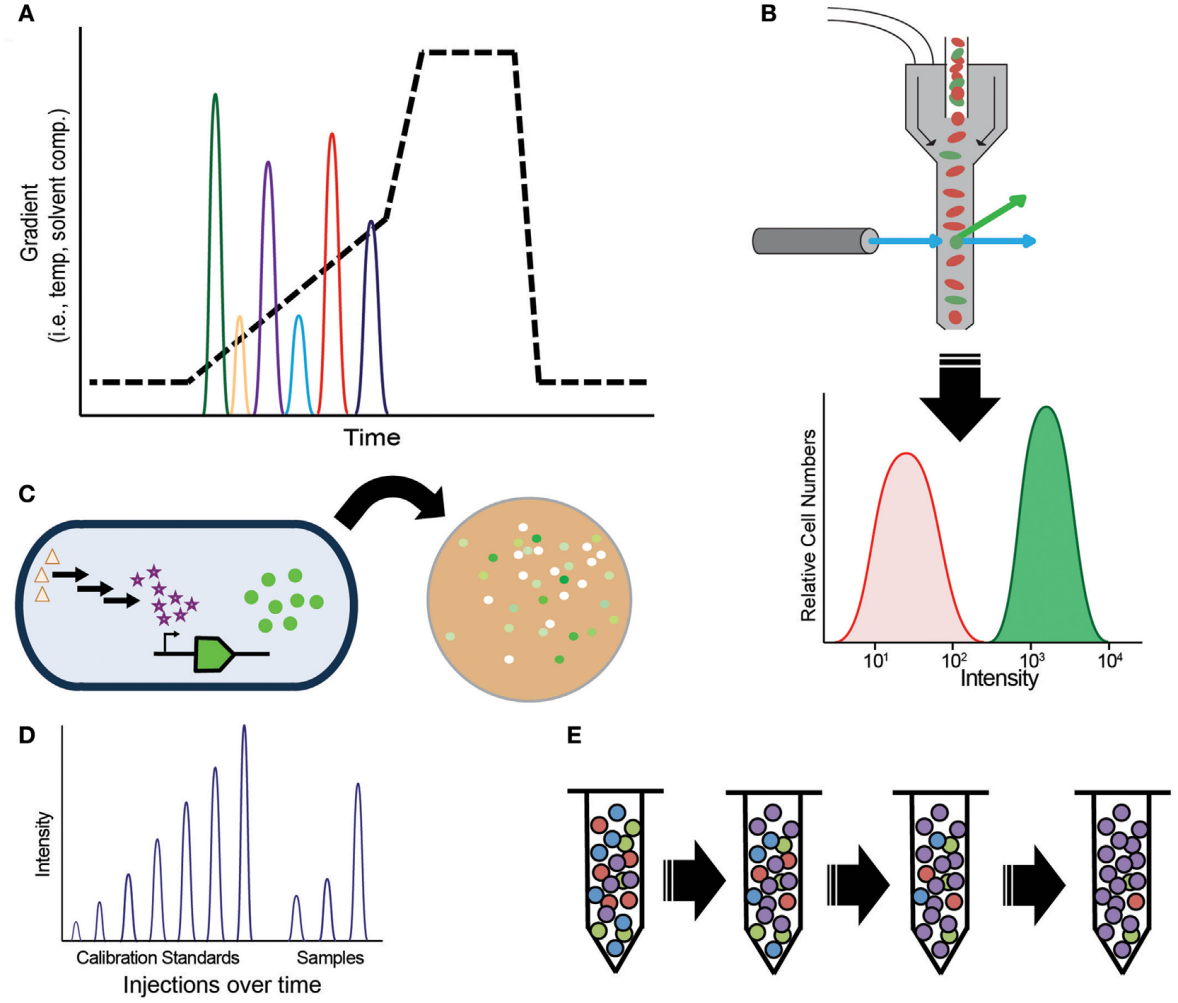
**Methods for target molecule measurements, (A) chromatography; (B) spectroscopy-based fluorescent-activated cell sorting (FACS); (C) biosensors; (D) direct injection mass spectrometry; (E) selection-based assays**.

By far the most common assay, and arguably the only necessary one, in metabolic engineering is target molecule detection. Target molecule detection takes several common forms that balance throughput and flexibility, with increased throughput typically coming at the cost of lower flexibility (Table 1). Small-scale engineering efforts often quantify the target of interest by using techniques such as gas or liquid chromatography (GC, LC) with UV absorbance or mass spectrometry (MS) detection (Figure 2A). The vast majority of older metabolic engineering studies relied heavily on these assays for initial pathway validation. Mass specific detection is particularly powerful because it permits monitoring the target molecule and, in many cases, pathway intermediates within complex matrices. Furthermore, assay development is typically fast and easy when standard compounds are available making them applicable to many types of targets. These methods produce confident target identifications with high sensitivity as well as accurate and precise quantification. There are many tools to generate rationally designed libraries or random strain diversity, which push the sample throughput capacity of these assays. More recently, these methods have been used to verify the top “hits” from high-throughput screening (HTS) assays.

**Table 1:** **Analytical attributes of common target molecule assay types**.

	Sample throughput (per day)	Sensitivity (LLOD)	Flexibility	Linear response	Dynamic range
Chromatography	10–100	mM	++	**+++**	**+++**
Direct mass spectrometry	100–1000	nM	**+++**	**+++**	++
Biosensors	1000–10,000	**pM**	+	+	+
Screens	1000–10,000	nM	+	++	++
Selection	**10^7^**^+^	nM	+	+	+

*The optimal method for a given criteria is highlighted in bold*.

While flexibility and confident identification are highly desired for proof-of-concept experiments, higher throughput assays, such as screens, selections, or biosensors, are preferred (Dietrich et al., 2010; Van Rossum et al., 2013) for titer, yield, and productivity optimization, steps required to develop economically viable strains. To achieve the necessary throughput, the vast majority of HTS assays rely on spectroscopic measurements, such as colorimetric, UV absorbance, or fluorescence in micro-well plates or via fluorescent-activated cell sorting (FACS) (Figure 2B). High-throughput measurements remain difficult for most target molecules because they lack an appropriate fluorophore, chromophore, or may not be essential for cell growth. A variety of chemical biology tools are available to overcome this complication by modifying target molecules with chemical tags. Bio-orthogonal chemistries, such as “Click” chemistry (Kolb et al., 2001), as well as protein bio-conjugation methods (Stephanopoulos and Francis, 2011) can be used to identify glycans, proteins, lipids, and nucleic acids. However, much effort is necessary to develop quantitative assays based on chemical biology tools. Ultimately, the usefulness of these assays depends on key performance features: dynamic range, sensitivity, and linear range of detection (Dietrich et al., 2010).

Most biosensors function via protein or transcript-based sensing of a target molecule coupled to some reporter (Figure 2C). By far, the most common use of biosensor assays is for spectroscopic measurement of the consumption or production of a cofactor, changes to pH, or H_2_O_2_. If gene expression as a response to target molecule production is known, then the expression of a reporter protein can be used. Recently, many enzymatic reporter systems have been generalized to broaden their applicability to new target molecules (Eggeling et al., 2015), but many lack suitable ligand recognition or binding elements. Consequently, engineering RNA aptamers (Win and Smolke, 2008; Babiskin and Smolke, 2011; Carothers et al., 2011; Michener et al., 2012; Zhang et al., 2012a), transcription factors (Binder et al., 2012; Dietrich et al., 2013), ligand binding proteins (Looger et al., 2003; Tang and Cirino, 2011; Shong and Collins, 2013), and protein–protein interactions (Dueber et al., 2003; Skerker et al., 2008) are fertile areas of research. Likewise, biosensors engineered to report via light/dark changes can be tuned to report on target production or various cell functions (Tabor et al., 2011). Whole cell biosensors that respond to target molecules via growth (Pfleger et al., 2006) effectively separate the reporter from the engineered microbe. These systems remove sensor components from the producer strain simplifying assay development and troubleshooting. By using synthetic transcription factors, one can produce metabolic enzyme sensors that recognize specific target molecules. Despite many examples of high-throughput assays in the literature, it is challenging to build a biosensor or screen with sufficient dynamic range, sensitivity, and linear response for a broad range of optimization conditions. To alleviate this constraint, more effort is needed to engineer genetic circuits that permit tight control of signal transduction, amplification, and response time (Kobayashi et al., 2004; Tabor et al., 2009).

Mass spectrometry is capable of the necessary throughput, sensitivity, and linear response for screening studies; however, signal repression from the complex matrix associated with cellular analyses confounds many MS assays, requiring slow separations prior to MS detection. New technologies, such as the Agilent Rapidfire™ system, attempt to overcome this limitation by integrating solid phase extraction with HT direct infusion analyses (Figure 2D) at a rate of one analysis per 10–12 s (one 96-well plate in (11 min). Initially applied to drug discovery studies (Vanderporten et al., 2013), there is a great interest in implementing the system to screen cell extracts for metabolic engineering research. This technique has the potential to determine target levels and comprehensively analyze multiple types of cell metabolites in much greater throughput than currently possible. Alternatively, development of less complex engineering hosts or cell-free systems would simplify MS measurements overcoming this obstacle.

Key to screen development is establishing conditions that produce accurate quantitative results that can be used to identify strains with improved production. Statistical methods, leveraged from drug discovery studies to verify hits, reduce false positives and false negatives (Malo et al., 2006, 2010) facilitate differentiation of subtle improvements. For industrial applications, the quality of the screen with respect to scale-up process requirements determines the ultimate success of the method. Consequently, development of these assays typically requires significant time, resources, and testable strains to ensure that quantitative improvements determined for micro-bioreactor conditions translate into analogous gains at production scale.

Despite the high-throughput nature of screening, there remains several bottlenecks that limit the number of strains that can be tested. Heavy reliance on colony picking and liquid handling systems produce a practical limit to throughput in addition to significant needs for culturing space. Selections, which circumvent these obstacles, are powerful methods to test very large libraries (10^10^ cells) to rapidly identify the optimal genotype (Figure 2E). Techniques such as TRMR (Warner et al., 2010) use selections to improve host tolerance and target production, yet few target molecules meet the criteria for selection-based assays (Dietrich et al., 2010). It can be very difficult to engineer a chosen host organism to be auxotrophic for a specific target molecule; consequently, there is a great interest in methods to adapt existing selections to new targets via biosensors. Biosensors combined with feedback-regulated evolution of phenotype (FREP) (Chou and Keasling, 2013) are a powerful way to evolve traits. Fluorescent-activated cell sorting of strains engineered with biosensor reporters offers significant increase in throughput without the associated culturing and colony-picking bottlenecks. Yet, variance is often broad requiring follow-up screens or chromatography-based assays to validate strain improvement. In most situations, achieving well-defined performance criteria for high-throughput assays is a long arduous process.

## Transcriptomic Analysis

Since the primary mechanism of change via metabolic engineering is DNA, many studies have focused on techniques and methods that reduce the cost of gene synthesis, strain construction, and other aspects of molecular biology. Through analysis of transcript levels and mapping the outcomes of failed systems, new constraints can be applied to successive iterations of the cycle. Traditional methods like real-time quantitative PCR (RT-qPCR) and microarray analyses are routinely used to verify that the host has been engineered correctly and to query regulatory and stress-related effects under production conditions. Combining microarray analysis with pathway intermediate detection, Kizer et al. (2008) identified stress response in *Escherichia coli* due to an imbalanced mevalonate pathway that accumulated 3-hydroxy-3-methylglutaryl-coenzyme A (HMG-CoA).

Transcript analysis has been particularly helpful for host-engineering efforts. Oh and Liao (2000) examined *E. coli* grown on glucose, acetate, and glycerol media and identified transcripts that were up- or down-regulated during protein overexpression. Alternatively, transcriptome analysis aided the identification of promoters responsive to specific growth conditions, such as the presence of inhibitors, pathway intermediates, and oxygenation state of the cell (Rutherford et al., 2010; Zhang et al., 2012a). For instance, to increase *E. coli* tolerance to the presence of ionic liquids microarray analysis was used to identify native *E. coli* promoters responsive to sub-lethal concentrations of ionic liquid (Frederix et al., 2014). Dynamic control of a newly discovered pump was engineered to alleviate ionic liquid toxicity. Designing dynamic control of pathway enzymes based on accumulation of pathway intermediates is greatly desired for commercial scale fermentations to eliminate the need for costly inducer compounds. Microarrays have also been used to identify promoters that respond to pathway intermediate accumulation. Amorpha-4,11-diene production in *E. coli* was improved by engineering pathway enzyme production in response to increased farnesyl pyrophosphate (FPP) levels (Dahl et al., 2013).

**Figure 3:**
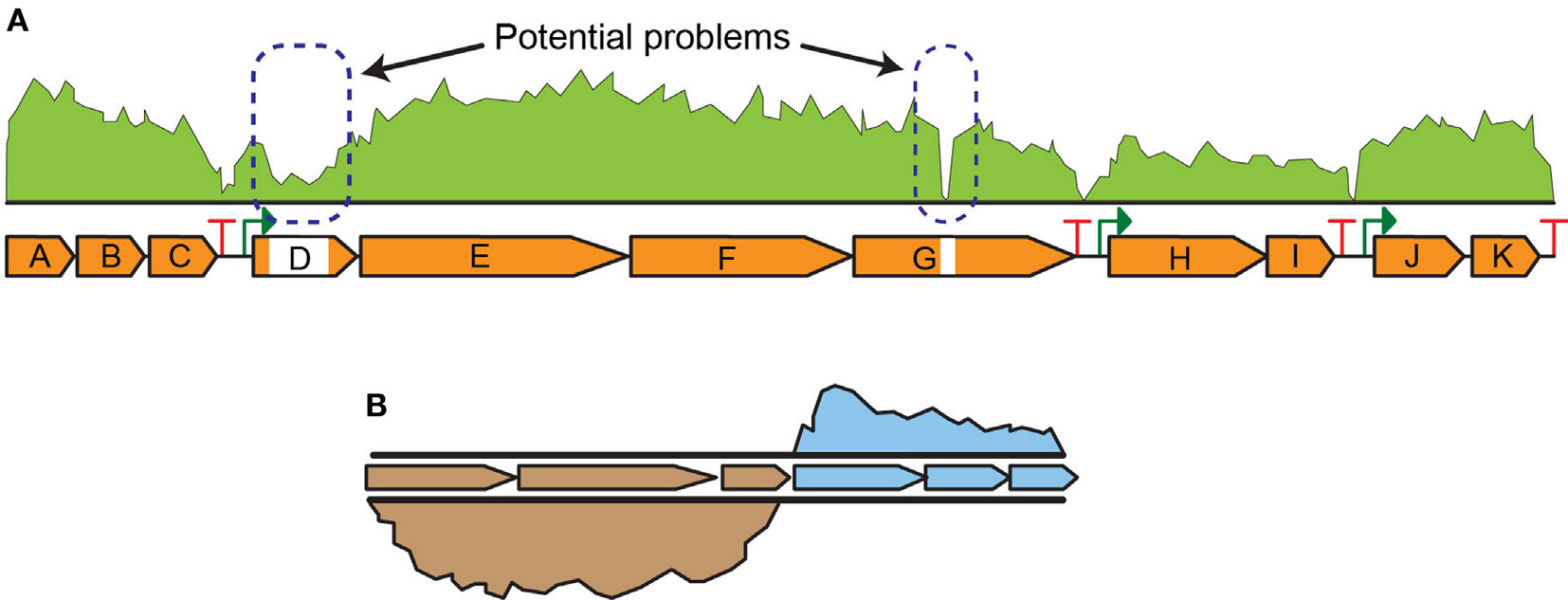
**Next-generation sequencing data examples for (A) engineered strain QA/QC indicating potential problems in transcript levels in parts of the pathway and (B) comparative RNA-seq analysis indicating higher expression of three genes in one strain relative to another strain**.

Next-generation sequencing (NGS) technologies (Smith et al., 2009) are emerging as the preferred approach for transcript analysis. NGS assays are suited to a broad range of applications, including quality assurance/quality control (QA/QC) of DNA construction efforts and quantitative whole genome transcript analysis (i.e., RNA-seq experiments) (Figure 3), due to high sensitivity, multiplex advantages, and dynamic range (Robles et al., 2012; Ghaffari et al., 2015). The most direct use of NGS is for QA/QC experiments of engineering pathways and strains. Tracking genomic changes during pathway and host engineering is crucial to determining the true (and testable) source of production improvements. NGS permits comprehensive validation of engineered strains to identify unintended mutations or other types of transcriptional failures (Figure 3A). For instance, identification of transcript read-through of terminators facilitated optimization of nitrogen fixation pathway in *E. coli* (Smanski et al., 2014). Genomic characterization of engineered strains via NGS is especially useful to rapidly identify single nucleotide changes that contribute to the observed phenotype. Comparative RNA-seq analysis was used to characterize the transcriptional response of *E. coli* to aromatic inhibitors from pretreated biomass and discover a d-galacturonic acid transporter candidate gene in *Neurospora crassa* that was then expressed in yeast (Benz et al., 2014). Similarly, it was used to identify xylose utilization genes and their regulation in *Saccharomyces cerevisiae* (Feng and Zhao, 2013). Additionally, RNA-seq is foundational to multi-omic analyses where integrated datasets are used to elucidate complex cellular interactions and identify differences between strains. Data generated from RNA-seq are also useful for genome-scale metabolic models (GSMs) as it is complementary to flux analysis (Gowen and Fong, 2010).

## Proteomic Analysis

Transcriptomic analysis readily queries the success or failure of strain construction efforts, yet it is often a poor proxy for protein abundance. Consequently, proteomic analysis is valuable for characterization of the functional aspects of engineered strains. Proteomic analysis is inherently more challenging than transcriptomic analysis because proteins are not readily amplified nor are they easily separated from each other; factors that dramatically impact sample throughput. Protein detection and quantification are frequently achieved via immunoblot assays because they are selective, established, fast, and easily analyzed in parallel. A variety of techniques are available for quantification of proteins via immunoblot assays or with fluorescent protein surrogates; however, these are also often inaccurate (Cardinale and Arkin, 2012). Furthermore, it can be challenging to obtain accurate quantitative information from these methods when assaying many different proteins in the same strain as is common for multi-step pathway engineering.

Current shotgun proteomic methods based on LC-MS/MS are useful for identification and quantification of thousands of proteins. Differential relative quantification is frequently achieved by culturing strains in media containing isotopically labeled substrates or by using functional group-specific chemical labels [e.g., iTRAQ (Shadforth et al., 2005), TMT (McAlister et al., 2012)] during sample preparation, or by so-called “label-free” techniques (Arike et al., 2012), such as data-independent analysis (DIA) (Gillet et al., 2012). Shotgun proteomic analyses are often combined with transcriptomic analyses to characterize cell stress responses to high levels of pathway intermediates or the final product (Rutherford et al., 2010) and to identify metabolic sinks that reduce carbon flux through the pathway.

**Figure 4:**
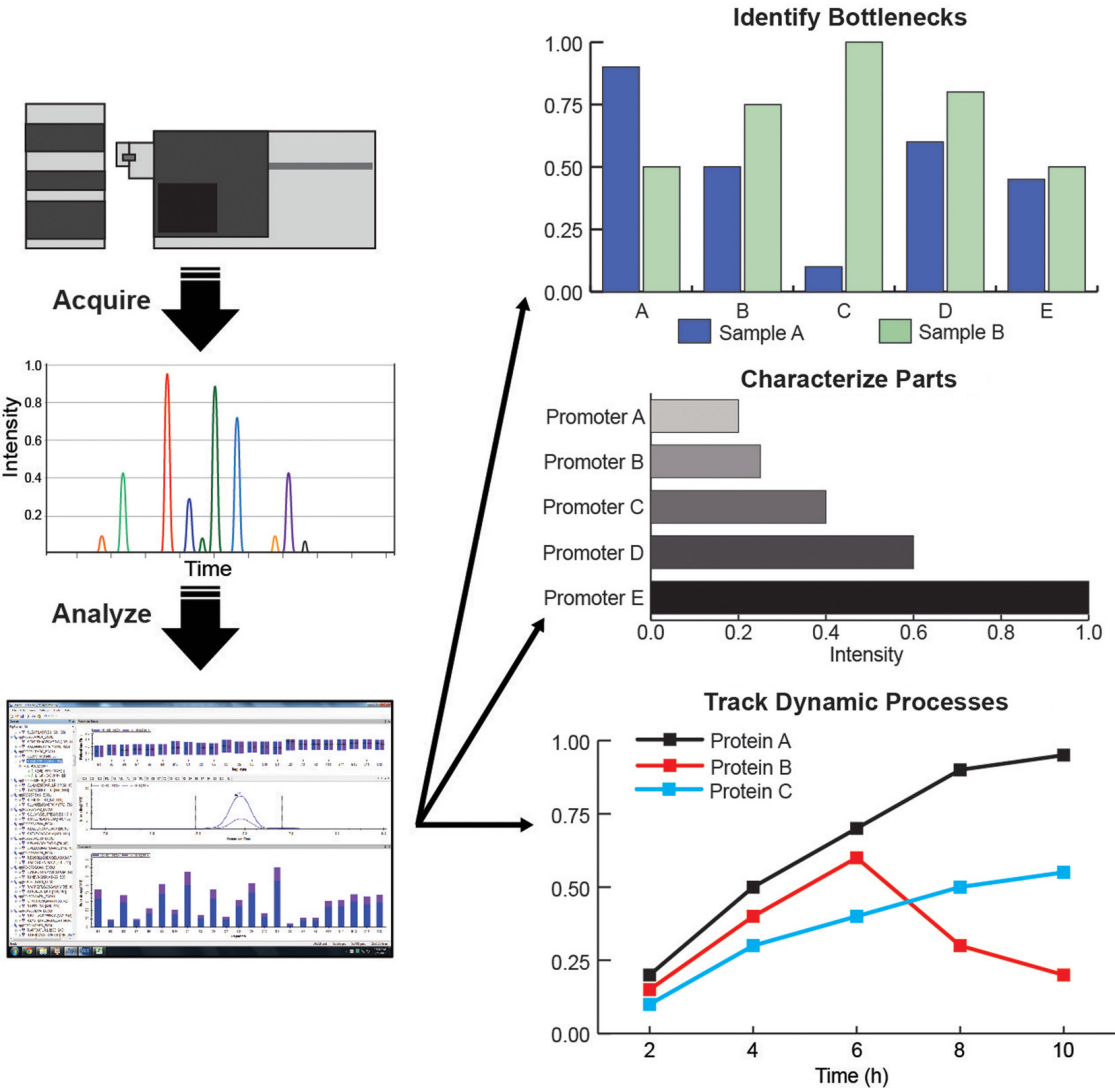
**(Left column) Targeted proteomic assay workflows: acquire data from LC/MS, curate data in Skyline, quantify and analyze protein levels and behavior; (Right column) Applications of targeted proteomics for metabolic engineering: identification of pathway bottlenecks, characterization of synthetic biology parts, and tracking dynamic processes**.

For more specific hypotheses, a targeted proteomics approach, via selected-reaction monitoring (SRM) MS can be used to accurately quantify a select group of proteins (Picotti et al., 2009; Picotti and Aebersold, 2012). The targeted proteomics approach has been applied in a variety of ways to test engineered microbes. Redding-Johanson et al. (2011) used targeted proteomics to identify protein-associated bottlenecks in the mevalonate pathway expressed in *E. coli*, resulting in over threefold improvement in the final product. This technique has been used to quantify protein levels for promoter and ribosome-binding site (RBS) variants (Nowroozi et al., 2014), comparison of enzyme homologs (Ma et al., 2011), and to track dynamic regulation of protein levels (Zhang et al., 2012b; Dahl et al., 2013) (Figure 4). More recently, targeted proteomic methods have been optimized for greater throughput (Batth et al., 2014) as well as used to characterize and quantify stable post-translational modifications for engineered microbes. For instance, on polyketide synthases (PKS), by targeting the active site peptide the degree to which it is modified by acyl precursors can be monitored via a phosphopantetheinyl-ejection assay (Dorrestein et al., 2006; Meier et al., 2011). Bottlenecks in PKS function can be determined from this assay similar to pathway metabolite analysis for modular pathways. It has been used to identify competing reactions and characterize the mechanism of PKS function (Hagen et al., 2014; Poust et al., 2015). Pairing bioinformatics tools to identify gene clusters with assays specific to PKS and NRPS enzymes will stimulate discovery of novel natural products (Bumpus et al., 2009). Adaptation of this technique to other PTMs will facilitate characterization of other classes of enzymes for metabolic engineering applications.

## Metabolite Analysis

Measuring protein levels in the cell goes a long way toward characterizing a microbial cell factory, yet, assuming that all of the protein is functional is often incorrect. Consequently, metabolite analyses at the pathway and organism level provide functional information for both pathway and host-engineering research. Tools for strain improvement, such as metabolic flux analysis and constraint-based reconstruction and analysis (COBRA), rely heavily on accurate metabolite data and carbon-flux measurements to restrict parameters for model predictions. Monitoring metabolites that are part the engineered pathway as well as central carbon metabolism aids identification of bottlenecks, and helps identify where increasing specific protein levels can yield dramatic improvements to the product titer or where allosteric regulation is limiting flux through the pathway. Metabolite analysis is commonly carried out as a part of GC-MS and LC-MS target molecule detection assays since pathway intermediates often have similar chemical structures to the target. Fortunately, LC-MS methods that were developed to study central metabolism (Bajad et al., 2006; Lu et al., 2008; Reaves and Rabinowitz, 2011) are directly applicable to metabolic engineering work. Various methods to quantify changes in secondary metabolites or specific pathway intermediates can be readily implemented by labs with the necessary instrumentation. Yet, developing assays for each pathway intermediate is often challenging due to the lack of available standards, intermediates that degrade rapidly, or ones that are isomers. These complications often result in incomplete information regarding the pathway metabolite levels. Designing specific methods for each pathway or class of target molecule is a time- and resource-consuming process that typically results in long LC-MS methods, severely limiting sample throughput. One way to circumvent low throughput LC-MS methods is by using flow-injection acquisition (FIA) (Fuhrer et al., 2011) that omits the chromatography step, relying on high mass accuracy, high-resolution MS for confident metabolite identification. FIA is heavily dependent on reproducible extraction and sample preparation conditions for quantification. A potential compromise between direct injection and long chromatographic methods is the combination of an on-line solid-phase extraction (SPE) method with direct MS detection (Vanderporten et al., 2013).

Non-targeted, discovery-based metabolomics experiments offer great opportunities for metabolic engineering based on comprehensive metabolome analysis. Extensions of genome-scale models to fully utilize the metabolome and integrate multiple omic data-types (Schellenberger et al., 2011; Lerman et al., 2012) have recently been developed to provide greater predictive power for engineered microbes. Coupling multiple extraction, sample preparation, and chromatography methods enables near complete characterization of the small molecule component of a microbe. Yet, sample complexity necessitates long chromatography gradients to adequately separate metabolites. Despite recent improvements in metabolite identification (Pan et al., 2011), non-targeted metabolomics is still a significant challenge requiring many control samples, metabolite standards, or extensive follow-up experiments. As a result, non-targeted metabolomics methods are most applicable for discovery experiments where the identity of the desired product is not well known, but are challenging to implement for high-throughput quantitative analyses.

## Developing Tools

**Figure 5:**
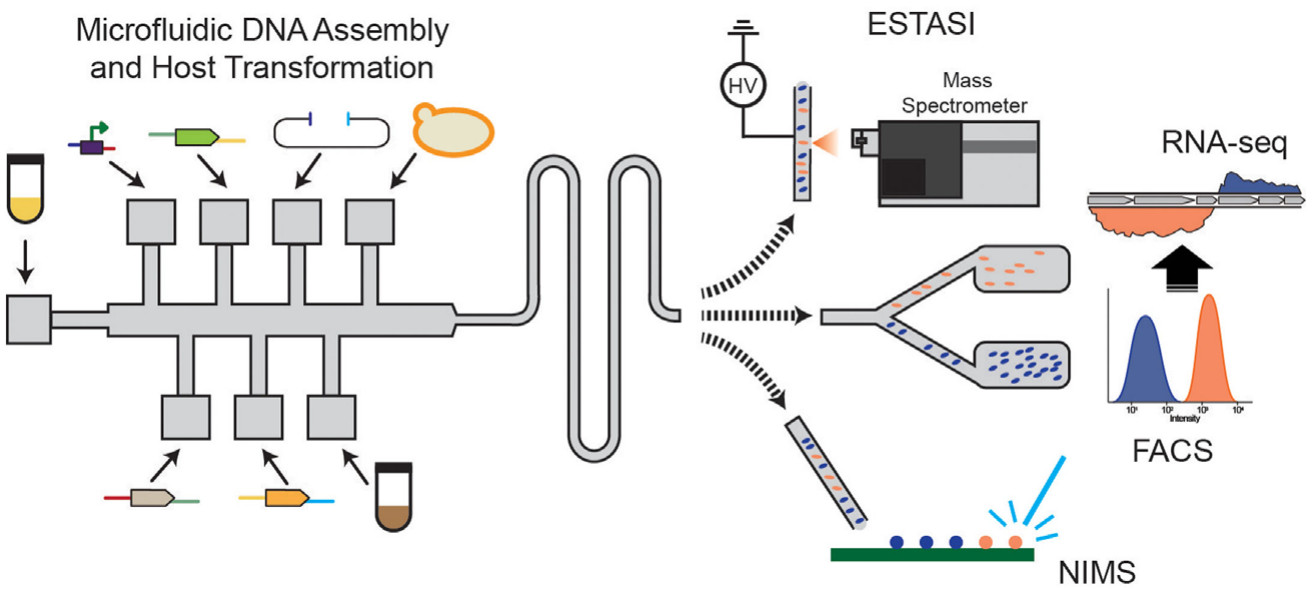
**Developing microfluidic assays for metabolic engineering with FACS-RNA-seq, nanostructure initiator mass spectrometry (NIMS), and electrostatic spray ionization (ESTASI) mass spectrometry analysis for high-throughput, small volume analysis**.

Beyond these technologies are new tools that have the potential to be catalysts for metabolic engineering research. Some of the most promising of these technologies are microfluidic (Liu and Singh, 2013; Wang et al., 2014; Shih et al., 2015) or droplet-based (Abate et al., 2013; Lim and Abate, 2013; Basova and Foret, 2015) systems to build, culture, and analyze many thousands of strains (Figure 5). These systems offer the tantalizing possibility of testing more than 10,000 unique genotypes per day while overcoming bottlenecks associated with colony picking and culturing limitations. Coupling microfluidic strain construction with FACS and single cell RNA-seq analysis (Abate et al., 2013; Saliba et al., 2014) holds great promise for ultra-high-throughput metabolic engineering. The strengths of these systems revolve around time and reagent cost savings associated with nanoliter and picoliter scale experiments coupled with sensitive spectroscopic assays. Expansion of the spectroscopic toolbox beyond UV/vis and fluorescence assays will depend heavily on adaptation of near-IR, mid-IR, and Raman-based methods that have been successfully implemented in other research fields (i.e., food science, health, materials science), but need further development to be robust for this scale.

There is also great interest in coupling microfluidic and droplet-based systems with mass spectrometric detection to broaden the type of information that can be produced in this manner (Figure 5). The prospective understanding that this level of strain characterization provides could be transformative. Attempts to couple direct infusion or desorption-based MS are underway and show promise (Kelly et al., 2009; Liu et al., 2010; Gao et al., 2013). For instance, a new ionization method, electrostatic spray ionization (ESTASI), that is compatible with droplet-based methods without dilution or an oil removal step demonstrated high sensitivity at 10 Hz sampling rate (Gasilova et al., 2014). However, issues associated with sample complexity, sensitivity, and acquisition time need to be overcome to enable broad application. The nanostructure initiator mass spectrometry (NIMS) assay combines the versatility of rapid MS analysis with specificity associated with initiators that can be synthesized for a given target (Northen et al., 2008) or are compatible with common chemical biology tools.

Complementing microfluidic and droplet-based systems are innovative assays based on cell-free systems and electrical current that yield very high-throughput advantages. Cell-free systems (Jewett et al., 2008; Hodgman and Jewett, 2012) simplify analysis due to lower complexity matrices, increase engineering flexibility, and enable rapid tuning of parameters, such as proteins or substrate levels (Wang et al., 2012). Similarly, engineering of the electron transfer pathway in *E. coli* provides the foundation for biosensor assays that produce electrical readouts (Jensen et al., 2010) to simplify and generalize detection. Likewise, new detection methods for biosensors based on carbon nanotubes and elemental-tag based antibodies have the potential to significantly broaden biosensor dynamic range and sensitivity (Selvaraju et al., 2008; Yang, 2012). Characterizing these methods for complex backgrounds, such as engineered microbes across a large target molecule concentration range, is necessary to establish robustness of the method and minimize false positives and false negatives.

Ribosomal profiling is an emerging method to quantify the fraction of mRNA transcripts that are being actively translated by ribosomes (Ingolia et al., 2009). This method offers the comprehensiveness of RNA-seq measurements with greater correlation to protein levels. Broad applicability is somewhat hindered by variable translation rates, variability in tRNA abundance, and technical challenges associated with polysome fractionation. Yet, all of these concerns are surmountable and with the widespread availability of NGS tools the potential to quantify actively translated protein is on verge of becoming a standard workflow in metabolic engineering research. Reverse phase protein arrays (RPPA) (Tibes et al., 2006) are an alternate protein quantification method that has the potential to greatly increase the throughput of proteomic data acquisition. Initial studies, focused on biomedical applications, indicate that it is very sensitive and reproducible but the biotechnology application landscape is unexplored. The time and resources required to develop antibodies for many protein targets and the subsequent experiments needed to validate the method result in slow progress. A large-scale investment for select industrial host proteins could establish a useful quantitative tool for many metabolic engineering applications.

An integrated omics platform has long been the goal of systems biology research across the health and biotechnology fields, yet challenges associated with low data quality, difficulty comparing different types of omics data, and a general lack of datasets for multiple omics methods have kept it from becoming reality. The recent advances to omics techniques described above relating to increased sample throughput, higher data quality, greater method robustness, along with their broader adoption, are encouraging from the data acquisition standpoint. Comprehensive datasets inform statistical analyses and rule-based approaches for subsequent designs that increase the success rate for achieving production of a target molecule. Computational tools, such as correlation analysis, PCA, and machine-learning methods, are just emerging and rely on accurate information to make quality predictions (George et al., 2014; Alonso-Gutierrez et al., 2015). And, development of tools to relate information regarding the genetic background as well as factors associated with cell culturing, sample preparation, acquisition, and analysis will be needed to extract the greatest benefit.

## Conclusion

The on-going cost and efficiency improvements occurring to the Design and Build components of the metabolic engineering cycle are placing significant strain on the Test component because many constructs are needed to find the optimal parameters. This presents a classic “chicken-or-the-egg” dilemma, whereby many test samples are needed to refine parameters for designs while well-defined parameters are needed to reduce the number of samples to test. Currently, a massive amount of resources must be directed to the development of analytical technologies to match the capabilities further upstream in the cycle with the intent to comprehensively cover the design space. Standardized procedures and data-type reporting for metabolic engineering test measurements will enable greater applicability of data across the field. Efforts focused on a combination of these analyses will yield detailed understanding of dynamic processes between multiple parts or systems. Efficient metabolic engineering will be possible with design-of-experiments methods to intelligently sample strains from large libraries of engineered strains. Large datasets, statistical analyses, and rule-based approaches for subsequent designs will inform models to increase the success rate for achieving production and optimization of a target molecule. In the end, with a rapidly turning DBTL cycle significant steps toward realizing the promise of metabolic engineering will be achievable.

## Conflict of Interest Statement

The authors declare that the research was conducted in the absence of any commercial or financial relationships that could be construed as a potential conflict of interest.
